# Reducing Relapse by Enhancing Reward Responsivity in Anorexia Nervosa: The VIBRANT (Virtual Interventions for Bolstering Recovery following Anorexia Nervosa Treatment) Trial ^†^

**DOI:** 10.20900/jpbs.20240005

**Published:** 2024-08-16

**Authors:** Ann F. Haynos, Kira G. Venables, Lisa M. Anderson, Michelle G. Craske, Carol B. Peterson

**Affiliations:** 1 Department of Psychology, Virginia Commonwealth University, Richmond, VA 23284, USA; 2 Department of Psychiatry, Virginia Commonwealth University, Richmond, VA 23284, USA; 3 Department of Psychiatry and Behavioral Sciences, University of Minnesota, Minneapolis, MN 55454, USA; 4 Department of Psychology, University of California, Los Angeles, CA 90095, USA; 5 Department of Psychiatry and Biobehavioral Sciences, University of California, Los Angeles, CA 90095, USA

**Keywords:** anorexia nervosa, treatment, reward, positive valence system, depression, anxiety

## Abstract

**Introduction:**

The post-acute phase of anorexia nervosa (AN) following discharge from higher-level care is a high-risk period in which relapse rates are high and many individuals lack access to effective treatment. Even after acute nutritional stabilization, AN is characterized by decreased biobehavioral sensitivity towards general rewards and elevated sensitivity towards weight-loss cues. These reward patterns may continue to maintain eating disorder and comorbid affective symptoms. To address these gaps in the treatment literature for post-acute AN, we propose a randomized controlled trial comparing Positive Affect Treatment for AN (PAT-AN), a neuroscience-informed therapy adapted to target these reward imbalances in AN, to more standard psychoeducational and behavioral treatment (PBT) for eating disorders following acute care.

**Method:**

Adult participants (*N* = 80) with broad AN, including atypical AN, discharged from intensive treatment (e.g., residential, partial hospitalization) for AN within the past 6 months will be randomly assigned to 24 weeks of remotely-delivered PAT-AN or PBT. We will compare the feasibility, acceptability, and efficacy of each treatment to augment post-acute outpatient care for AN. A multimodal neurocognitive and self-report battery will assess eating pathology, comorbid symptom, and putative reward mechanism changes over the course of treatment (i.e., baseline, mid-treatment, post-treatment, three-month follow-up) and on a week-to-week basis.

**Discussion:**

This trial will, for the first time, directly target observed reward disturbances in the post-acute period of AN. Thus, this investigation has the potential to simultaneously evaluate a novel, efficacious treatment for AN and to further evaluate the role of reward dysfunction in AN maintenance.

## INTRODUCTION

Anorexia nervosa (AN) is a serious illness characterized by a persistent drive towards weight loss leading to starvation [1]. AN is associated with severe physical and psychological morbidity [2] and among the highest mortality rates of any psychiatric disorder [3]. Although intensive treatment (e.g., residential treatment) may temporarily improve weight and eating habits [4], up to 50% of individuals with AN relapse in the year following discharge from higher-level care [5]. Thus, the post-acute period of AN following initial eating and weight stabilization constitutes a high-risk time in the clinical course of this disorder.

Full recovery from AN requires improvements in physical, behavioral, and psychological functioning [6], yet weight restoration often solely determines discharge from intensive care [7], limiting the ability to achieve full clinical improvement in these settings. Following acute care for AN, many individuals lack access to specialist treatment, receive inadequate aftercare, and return to environments that reinforce eating disorder symptoms [8,9]. Even among those who receive specialist care, there is limited guidance about how to treat post-acute AN. A recent systematic review identified only 14 clinical trials in the past 35 years targeting post-acute AN [10]. On average, these studies reported small-to-negligible effects on weight, eating disorder symptoms, or comorbidities. Thus, there is an urgent need to develop effective, accessible treatments for the post-acute phase of AN.

In addition, most existing interventions for AN across illness stages do not adequately target comorbid affective symptoms. Comorbidity between AN and mood and anxiety disorders is high [11] and individuals with AN often maintain affective disorders for decades after intensive care [12]. Extant treatments for AN typically do not substantially improve mood or anxiety symptoms [13], which may contribute to the high relapse rates in this population. Depression and anxiety are associated with poorer eating disorder treatment outcomes [14,15] and a recent meta-analysis found that comorbid depression predicted eating disorder relapse [16]. Novel treatment approaches that target shared mechanisms between AN and affective disorders hold promise for decreasing comorbid symptoms alongside eating disorder symptoms and promoting lasting recovery.

Dysregulation of the appetitive neurobiological reward system is one set of mechanisms implicated in both AN and affective disorders [17,18]. Evidence from multiple biobehavioral methods converges to indicate that individuals with AN, similar to individuals with affective disorders [17], demonstrate impaired reward anticipation, experiencing, and learning towards a range of typically rewarding stimuli, such as palatable food, social experiences, and monetary gains [19,20]. This research suggests that individuals with AN report less desire for and enjoyment of these experiences, as well as dampened engagement of the brain’s reward circuitry in response to typically-rewarding cues (e.g., winning money). Individuals with AN have also demonstrated over-activation of cognitive control brain regions during reward anticipation and receipt, indicating a tendency to down-regulate responses to positive stimuli [21,22]. However, paradoxically, AN is also associated with elevated reward responsivity towards weight-loss-specific cues (e.g., low-calorie foods, underweight bodies) [19], distinguishing AN from other disorders that are characterized by decreased reward sensitivity across contexts [23]. Evidence suggests that such dysfunctional biobehavioral reward processes may perpetuate the AN symptoms, as positive affect enhancement has been identified as a primary function of restrictive eating [24].

We have proposed a model that posits that, because individuals with AN struggle to find reward everyday positive experiences (i.e., social interaction, leisure activities), but readily experience reward from eating disorder symptoms, they may increasingly turn to such symptoms as a means of temporarily alleviating anhedonia and experiencing positive mood [25]. Over time, this process may increase the drive to engage in disordered eating and heighten the reward salience of eating disorder cues, maintaining eating disorder symptoms. At the same time, overreliance on eating disorders symptoms to derive reward may further decrease the salience of and approach towards non-eating disorder rewards, perpetuating affective symptoms ([25] provides a more detailed description of this theory). Although knowledge of the neurobiology of reward in AN has drastically increased over recent years, treatments targeting neuroscience-derived reward mechanisms are lacking, potentially limiting the ability to promote lasting change for these disorders [26].

In order to target shared neurobiological reward mechanisms perpetuating eating disorder and affective symptoms in AN, our group recently adapted and piloted a novel neuroscience-informed treatment, Positive Affect Treatment (PAT), for suitability in AN. PAT was originally designed to target the neurobiological reward disturbances underling anhedonia in mood and anxiety disorders [17]. Among individuals with depressive and anxiety disorders, initial trials of PAT have been associated with improvements in positive and negative affect, depressive symptoms, anxiety symptoms, suicidality, and mechanistic targets of reward motivation and responsivity [27,28].

Given the potential mechanistic overlap in reward disturbances between affective disorders and AN, we retained the core targets and structure of PAT in our adaptation, PAT-AN, while adding complementary interventions to reduce or replace eating disorder-relevant reward responsivity [25]. We recently conducted a small, randomized, waitlist-controlled pilot study of PAT-AN (*n* = 20) [29]. Preliminary data from this trial suggested that this treatment was highly feasible and acceptable, with 100% retention in the PAT-AN group, and demonstrated initial evidence of efficacy for improving eating disorder, mood, and anxiety symptoms that largely persisted at a 3-month post-treatment follow-up. Findings regarding the degree to which PAT-AN altered the self-report of positive affect and the anticipation and experiencing of general, social, and eating disorder-specific rewards was inconclusive, with some indices improving, some not changing, and others worsening following treatment (see Supplement A for further details). These results suggested the need for more comprehensive, multimodal assessment of reward functioning expanding beyond retrospective self-report to more objective or in-the-moment methods (e.g., neurocognitive and ecological momentary assessment), allowing for a triangulation of results from subjective, objective, in-lab, and real-world measures.

### Current Study: The Virtual Interventions for Bolstering Recovery following Anorexia Nervosa Treatment (VIBRANT) Trial

To extend our prior research, we propose a new study entitled, “Virtual Interventions for Bolstering Recovery following Anorexia Nervosa Treatment” (i.e., the VIBRANT trial). This protocol will involve a larger randomized controlled trial (*N* = 80) that will target reward disturbances in the high-risk period of AN following higher-level care discharge. Additionally, we will expand the reach of PAT-AN through utilization of remotely-delivered therapy. We will compare the efficacy of PAT-AN to more standard psychoeducation and behavioral treatment (PBT) for eating disorders (both remotely-delivered) in augmenting post-acute outpatient care for AN. We will also conduct a thorough, neuroscience-informed assessment battery of the putative reward processes targeted in AN to inform further mechanistic and treatment development. This will improve the ability to detect alterations in reward responsivity following treatment by employing methods that do not rely on self-report (i.e., neurocognitive assessment) or retrospective recall (i.e., ecological momentary assessment). Specific study aims include:

Aim 1: To examine the efficacy of PAT-AN compared to PBT for post-acute AN. We hypothesize that, compared to PBT, PAT-AN will demonstrate higher acceptability, lower attrition, and improved eating disorder and comorbid symptom outcomes (e.g., depression, anxiety, suicidality) at end-of-treatment and follow-up.

Aim 2: To examine the target engagement of putative reward mechanisms by PAT-AN compared to PBT for post-acute AN. We hypothesize that, compared to PBT, PAT-AN will exhibit increases in general reward processing and decreases in weight-loss-related reward processing at end-of-treatment and follow-up.

Aim 3: To determine if clinical change in post-acute AN results from the engagement of reward targets. Across groups, we hypothesize that increases in global reward sensitivity and decreases in weight-loss-related reward sensitivity will predict decreases in eating disorder and comorbid symptoms.

## MATERIALS AND METHODS

### Participants

We will recruit 80 adults with broadly-defined AN, including AN and atypical AN [1], who are within 6 months of discharge from intensive care (i.e., inpatient, residential, partial hospitalization, or intensive outpatient treatment) for AN. Atypical AN will be included given the mounting evidence suggesting that AN and atypical AN are highly similar in symptom presentation and severity [30]. However, it should be noted that there are limitations to the inclusion of atypical AN (see Conclusions and Limitations section below). Inclusion criteria include: (1) ≥18 years old; (2) English-speaking; (3) diagnosis of AN with body mass index (BMI) < 18.5 kg/m^2^ or atypical AN with loss of ≥10% body weight at higher-level care admission (to establish sufficient low weight/weight loss for diagnosis) and a current BMI ≥ 17.5 kg/m^2^ (to ensure a medically safe weight for outpatient treatment); (4) access to a smartphone or computer; and (5) willingness to participate in weekly assessments and to identify a primary healthcare provider. Participants with AN will also be required to have demonstrated an increase of ≥0.5 kg/m^2^ during acute care as evidence that they are appropriate for post-acute care. Exclusion criteria include: (1) acute medical instability; (2) pregnancy; (3) substance use disorder within the past 3 months; (4) lifetime primary psychotic or bipolar-I disorder; and 5) enrollment in treatment highly overlapping with PAT-AN. Supplement B describes the inclusion/exclusion criteria rationale. Recruitment will occur through print and online advertisements at eating disorder clinics, campus and community locations, and social media.

### Procedures

Assessment and treatment procedures will occur remotely. Interested individuals will complete pre-screening questionnaires through a recruitment registry. Potentially eligible participants will be invited to attend 1–2 screening visits consisting of informed consent, clinical and mechanistic assessments, and medical screening to determine eligibility and clinical baseline. Eligible participants will be randomized to PAT or PBT and then will proceed with treatment sessions. Although blinding of therapists and participants to study condition will not be possible, study assessors will remain blind to condition to ensure that assessment results remain unbiased. Supplement C outlines procedures for establishing and monitoring medical stability at baseline and throughout treatment.

To evaluate symptom and mechanistic changes across time, participants will complete a set of interviews, questionnaires, and neurocognitive tasks at four virtual visits: (1) baseline, (2) mid-treatment, (3) end-of-treatment, and (4) three-month follow-up. One week of ecological momentary assessment (EMA) will be delivered to participants’ cell phones to assess real-world symptoms and reward targets at baseline and end-of-treatment. Participants will be compensated for each major study visit, with bonus compensation available for high EMA compliance (responding to >75% of prompts).

### Treatment

Randomization to PAT-AN (*n* = 40) or PBT (*n* = 40) will be stratified by AN subtype at higher-level care admission (AN restrictive subtype, AN binge-purge subtype, or atypical AN) and level of care (inpatient/residential/PHP/IOP) using a computer algorithm. Both PAT and PBT are 50-minute individual treatments that will be delivered remotely through secure video conferencing weekly for 24 weeks. These treatments will be delivered adjunctive to treatment as usual (including no other treatments). Prior to each session, participants will have blind weight measured and complete pre-session questionnaires assessing eating and mood symptoms. Following each session, participants will complete post-session questionnaires assessing treatment acceptability.

Therapists will be required to have masters-level or higher education. Therapists will be trained in the study treatments during two half-day workshops (one dedicated to each study treatment), with annual half-day refresher trainings thereafter. Therapists will all participate in weekly one-hour group supervision meetings, which will include a safety monitoring, review of cases with clinical and adherence feedback, and a review of session video or audio recordings for treatment fidelity.

#### Positive affect treatment for Anorexia Nervosa (PAT-AN)

PAT-AN is an adaptation of PAT, a neuroscience-informed psychosocial therapy designed to target anhedonia by enhancing reward anticipation, experiencing, and learning through a combination of cognitive, behavioral, mindfulness, and self-compassion techniques [17,28]. In PAT-AN, additional interventions have been added to reduce or replace any positive affect modulating functions of eating disorder behavior. The content of PAT-AN and adaptations for AN have been described in detail elsewhere [25]. Following the initial pilot study, additional updates were made to accommodate therapist and participant feedback, including: (1) further extending treatment length to 24 weeks; (2) enhancing the concreteness of experiential exercises; and (3) more explicitly weaving a focus on eating disorder-related challenges into treatment exercises. The current version of PAT-AN consists of six modules targeting common biobehavioral reward disturbances in AN (Table 1).

#### Psychoeducation and behavioral treatment (PBT)

The treatment comparator is based on educational and behavioral comparison conditions in prior trials [31,32] and common elements of standard eating disorder treatments [33,34], including collaborative weighing, food monitoring, establishing regular eating patterns, and food- and body-related exposures. PBT was carefully designed to avoid direct targeting of reward responsivity to maintain clear distinctions between treatments; however, the treatment is structured to parallel the modular format of PAT-AN (Table 1).

### Measures

Table 2 provides an overview of the study assessment schedule. Supplement D provides expanded details about study measures.

#### Demographics and eligibility

Age, race/ethnicity, sex/gender, and additional demographic characteristics will be collected at baseline to characterize the sample and ensure adequate matching between conditions. The Mini International Neuropsychiatric Interview [40] will determine baseline psychiatric diagnoses and eligibility.

#### Feasibility, acceptability, and satisfaction

Treatment completion, measured both dichotomously (i.e., treatment completer vs. non-completer) and continuously (i.e., number of sessions completed) will serve as a measure of treatment acceptability. Further, at end of treatment, participants will complete two measures adapted from our prior trials (e.g., [35]), the Treatment Acceptability, Feasibility, and Satisfaction Questionnaire (TAFSQ) and the Component Acceptability Questionnaire, to rate the acceptability of various treatment components.

#### Eating disorder symptoms

BMI will be calculated at all major study visits and weekly prior to study sessions. At baseline, participants will be mailed a tape measure to measure objective height and a blind smartscale (e.g., MyClearStep^™^) to measure objective weight. Both measurements will be conducted using standardized procedures (e.g., shoes and outer garments removed) and supervised by research staff over a video call. The smartscale will transmit weight measurements to the research team without the participant being able to view them.

The Eating Disorder Examination (EDE) [39] will be administered at all major study visits and the EDE Global score will be the primary measure of eating disorder symptoms. The Change in Eating Disorder Symptoms Scale (CHEDS) [36] questionnaire will be administered weekly to assess fine-grained changes in eating disorder symptoms throughout treatment.

#### Affective symptoms

Depression and anxiety symptoms will be assessed with the Depression, Anxiety, and Stress Scale (DASS-21) [38] at major study visits. An abbreviated version (DASS-9) [44] will be administered before sessions to capture week-to-week depression and anxiety changes. The Columbia Suicide Severity Rating Scale (C-SSRS) [37] semi-structured interview will assess suicidality at major study visits and the briefer, 6-item questionnaire version will be administered weekly to assess fine-grained suicidality changes throughout treatment.

#### Reward measures

General reward sensitivity will be assessed using the positive affect subscale of the Positive and Negative Affect Schedule (PANAS) [41] and the Temporal Experiences of Pleasure Scale (TEPS) [43]. Eating-disorder-specific reward sensitivity will be measured using the positive subscale of the Pros and Cons of Anorexia Nervosa Scale (PCAN) [42]. Week-to-week positive affect changes will be assessed using PANAS positive subscale scores.

#### Neurocognitive tasks

Participants will complete two neurocognitive tasks at baseline, mid-treatment, and end-of-treatment to measure reward-based decision-making in general and weight-control contexts.

The WebSurf Task [45] is a reward-based decision-making paradigm. Participants “surf” through different video galleries (i.e., animals, art, landscapes, dance) for a fixed amount of time. The participant is presented with a random time delay (3–30s) to watch the video and must decide whether to stay through the delay or skip to the next gallery for a new choice. When the participant chooses to stay, they watch a brief clip and rate the enjoyment of the video. Delay “threshold” (i.e., the amount of time participants are willing to wait) and video enjoyment ratings will assess general reward anticipation and experiencing, respectively.

The Food Choice Task [46] measures reward-based decision-making to restrictive eating cues. Participants rate the healthiness and tastiness of a series of high- and low-fat foods, and then choose the item they would prefer to consume between the same foods and a reference food rated “Neutral” on health and taste. Low-fat food choice and tastiness ratings will assess weight-loss specific reward anticipation and experiencing, respectively.

#### Ecological momentary assessment

Participants will complete one week of EMA at baseline and end of treatment using procedures from prior research [35,47]. Positive affect changes (measured via the PANAS) associated with positive life events (e.g., social, leisure, mastery activities) and weight-control behaviors (e.g., restrictive eating, driven exercise) will be measured to assess general and weight-control specific reward (see Supplement E for full EMA protocol).

#### Statistical analyses

Supplement F details study power analyses and missing data treatment. For all mixed effects models, subject ID will serve as a random effect and treatment group, time, and group-by-time interactions will be fixed effects. Other random effects structures (e.g., random slopes) will be explored to determine if they improve model fit. We will run analyses both with AN and atypical AN groups combined (primary) and separated (exploratory) to determine if treatment effects differ between these diagnostic groups. The Benjamini-Hochberg procedure [48] will correct for familywise alpha for each set of primary analyses. For exploratory analyses, *p* < 0.05 will be used.

#### Analytic plan

Aim 1: To examine the efficacy of PAT-AN compared to PBT for post-acute AN. To test for feasibility and acceptability differences, primary analyses will compare completion (attending >80% of sessions) and non-completion between the treatment groups. Logistic and linear regressions will compare groups on dropout at end-of-treatment (primary) and follow-up (secondary), as well as number of sessions completed. A generalized linear mixed model will compare treatment groups on attrition over time. Linear regressions will compare treatment groups on acceptability on end-of-treatment TAFSQ scores.

To test impact of the treatments on eating disorder and affective symptoms, linear mixed effects models will compare groups on primary (BMI, EDE Global symptoms) and secondary (DASS-21 depressive symptoms and anxiety symptoms, C-SSRS suicidality) outcomes at major assessment points (baseline, mid-treatment, end of treatment, and follow-up). Post hoc analyses will compare treatment groups at each time point. Secondary analyses will include linear mixed effects models to compare groups on BMI, eating disorder symptom (CHEDS), and depression and anxiety (DASS-9) trajectories using weekly ratings. We will exploratorily examine if treatment effects are moderated by any of the measured reward indices.

Aim 2: To examine the target engagement of putative reward mechanisms by PAT-AN compared to PBT for post-acute AN. To examine differences in putative reward targeting with each treatment, linear mixed effects models will compare treatments on general and eating-disorder specific reward sensitivity (measured by the self-report, EMA, neurocognitive measures) at all major assessment points (baseline, mid-treatment, end of treatment, and follow-up). Post hoc analyses will compare treatment groups at each time point. Secondary analyses will include similar linear mixed effects models to compare groups on trajectories of PANAS positive affect scores using weekly ratings.

Aim 3: To determine if clinical change in post-acute AN results from the engagement of reward targets. Cross-lagged linear effects mixed models will examine relations between general and weight-loss specific reward sensitivity measures and primary and secondary symptom measures at the subsequent time point (i.e., baseline reward measures predicting end-of-treatment symptoms; end-of-treatment reward measures predicting follow-up symptoms). Exploratory mediation analyses will examine indirect (mediating) effects of each reward sensitivity metric on the relations between the treatment group and primary and secondary outcomes.

## CONCLUSIONS AND LIMITATIONS

PAT-AN is a neuroscience-informed treatment adapted to target the reward dysregulation hypothesized to maintain AN. Initial data suggest that this treatment holds promise for improving eating disorder symptoms, comorbid affective symptoms, and reward processing. We propose a randomized controlled trial comparing the efficacy of PAT-AN to PBT in augmenting recovery during the post-acute phase of AN, a vulnerable period for relapse. We will investigate treatment impacts on feasibility, acceptability, eating disorder and comorbid symptoms, and presumed reward mechanisms.

This investigation has a number of strengths, including investigation of a novel treatment and mechanistic targets, rigorous clinical trial design (i.e., randomization, active control condition, blind assessment), and innovative multimodal mechanistic assessment battery. However, there are also limitations to the study design that warrant consideration. First, participants will be permitted to engage in other types of outpatient treatment that are non-overlapping with PAT-AN. This decision was made for ethical reasons (due to PAT-AN being a new, relatively untested treatment) and in an effort to parallel treatment as usual following higher-level care, which often involves multidisciplinary interventions [4]. However, these outside treatments add uncontrolled variability that could impact the ability to detect between-condition differences. Further, it is possible that clinical improvements noted in either condition could result from outside interventions. To mitigate these concerns, the research team will measure treatment utilization outside of the study and examine this as an exploratory covariate. Second, although remote treatment via videoconferencing has become increasingly common [49], this delivery format remains largely untested, which may impact study results. Thus, future research will be required to determine if PAT-AN effects vary by treatment modality. Third, the inclusion of participants with atypical AN may add a level of heterogeneity to the study sample that could impact study findings. Although a growing body of research has highlighted similarities between AN and atypical AN [30], some research suggests that these disorders have may differ in certain mechanistic processes, including their genetic and metabolic underpinnings [50]. Thus, there are disparate opinions about whether these diagnoses should be collapsed or separated [51,52]. For this reason, we will stratify randomization according to diagnosis and conduct exploratory subgroup analyses to determine if treatment effects vary by group.

Despite these potential limitations, this investigation has the potential to both evaluate a novel and efficacious treatment for a critical stage of illness, and to further establish the role of reward dysfunction in the maintenance of AN. Thus, the results of this study hold promise to advance both mechanistic and treatment research for this severe, persistent, and life-threatening disorder.

## Supplementary Material

Supplementary files A-F

## Figures and Tables

**Table 1.** Overview of Positive Affect Treatment and Psychoeducation and Behavior Treatment modules and content.

	Positive Affect Treatment (PAT)		Psychoeducation and Behavior Treatment (PBT)	

Module number	Module Name	Content	Module Name	Content
1	Psychoeducation (Sessions 1–2)	Psychoeducation regarding positive mood, the components of mood, and the effects of low positive mood, highlighting links between positive mood and eating disorder symptoms in post-acute AN.	Psychoeducation (Sessions 1–2)	Psychoeducation regarding eating disorder symptoms in post-acute AN, including the cognitive-behavioral model of eating disorder symptoms, highlighting the self-perpetuating nature of symptoms and risk factors for relapse.
2	Developing the Positive (Sessions 3–7)	Planning and monitoring activities to generate short- and long-term positive mood, with a focus on trying new activities and using activities to prevent or delay eating disorder behaviors.	Stabilizing Eating Patterns (Sessions 3–7)	Behavioral techniques to establish and maintain a regular pattern of eating through collaborative weighing, self-monitoring of food intake, and planned meals.
3	Attending to the Positive (Sessions 8–12)	Cognitive and behavioral strategies for increasing positive mood through noticing positive aspects of situations, taking ownership of contributions to positive events, and anticipating future positive situations.	Decreasing Avoidance and Preoccupation (Sessions 8–12)	Cognitive and behavioral strategies for reducing weight- and shape- avoidance and hyper-monitoring through planned exposure and cognitive restructuring.
4	Cultivating the Positive (Sessions 13–17)	Cultivating positive emotions through mindfulness skills, with a focus on directing these skills towards the self and recovery.	Breaking Dietary Rules (Sessions 13–17)	Reducing dietary restriction through self-monitoring and dietary planning designed to break dietary rules.
5	Shifting the Positive (Sessions 18–23)	Identifying rewarding aspects of the eating disorder and learning to shift away from eating disorder behaviors and towards safer alternatives, particularly in difficult eating- and body-related situations.	Anticipating High-Risk Situations (Sessions 18–23)	Using problem-solving skills to identify and plan for high-risk situations for engaging in eating disorder behaviors.
6	Relapse Prevention (Session 24)	Relapse prevention and creating a plan for continued use of PAT strategies.	Relapse Prevention (Session 24)	Relapse prevention and creating a plan for continued use of PBT strategies.

Note: AN = anorexia nervosa; PAT = positive affect treatment; PBT = psychoeducation and behavioral treatment.

**Table 2.** VIBRANT trial assessment schedule.

Measure	Week					
	0 Weeks (Baseline)	1–11 (Weekly)	12 (Mid-Treatment)	13–23 (Weekly)	24 (EOT)	36 (Follow-up)
***Eligibility/Other***						
MINI	X					
Demographics	X					
***Treatment Acceptability***						
TAFSQ			X		X	
CAQ			X		X	
***Outcome Measures***						
EDE	X		X		X	X
BMI	X	X	X	X	X	X
CHEDS	X	X	X	X	X	X
DASS	X	X	X	X	X	X
C-SSRS	X	X	X	X	X	X
***Mechanistic Measures***						
PANAS	X	X	X	X	X	X
PCAN	X		X		X	
WebSurf Task	X		X		X	
Food Choice Task	X		X		X	
EMA	X					

Note: CAQ = Component Acceptability Questionnaire [35]; CHEDS = Change in Eating Disorder Symptom scale [36]; C-SSRS = Columbia Suicide Severity Rating Scale [37]; DASS = Depression, Anxiety, and Stress Scale [38]; EDE = Eating Disorder Examination [39]; EMA = Ecological momentary assessment; EOT = End-of-treatment; MINI = Mini International Neuropsychiatric Interview [40]; PANAS = Positive and Negative Affect Schedule [41]; PCAN = Pros and Cons of Anorexia Nervosa Scale [42]; TAFSQ = Treatment Acceptability, Feasibility, and Satisfaction Questionnaire [35]; TEPS = Temporal Experiences of Pleasure Scale [43].

## Data Availability

The dataset generated from the study will be made available through the National Institute of Mental Health Data Archive.
