# Supplementary material for: Reducing Relapse by Enhancing Reward Responsivity in Anorexia Nervosa: The VIBRANT (Virtual Interventions for Bolstering Recovery following Anorexia Nervosa Treatment) Trial
Source: J Psychiatr Brain Sci. Author manuscript; Available in PMC 2024 Nov 26. (PMC11583181; doi:10.20900/jpbs.20240005)
Supplement: Supplementary files A-F [file NIHMS2017322-supplement-Supplementary__files_A-F.pdf]

SUPPLEMENTAL MATERIAL

A. Expanded Description of Reward Measure Findings from PAT-AN Pilot Study

In our pilot study of Positive Affect Treatment for Anorexia Nervosa (PAT-AN), general reward anticipation and experiencing were assessed using the anticipatory and consummatory subscales of the Temporal Experience of Pleasure Scale (TEPS; [43]), social reward sensitivity was assessed using the Revised Social Anhedonia Scale (RSAS; [53]), and eating disorder-related reward was assessed using the Pros subscale of the Pros and Cons of Anorexia Nervosa Scale (PCAN; [42]). Weekly changes in positive affect throughout treatment were assessed using the Positive subscale of the Positive and Negative Affect Schedule (PANAS; [41]).

Results from the pilot showed that session-level positive affect did not significantly increase from pre- to post- treatment ($B = 0.09$, $SE = 0.05$, $p = 0.072$) during PAT-AN. However, participants in the PAT-AN group displayed small to large pre- to post- treatment improvements for PANAS positive, TEPS anticipatory, and RSAS social reward scores ($ds = 0.47$–0.86). Improvements in PANAS positive affect and RSAS social reward were sustained at follow-up, while post-treatment and follow-up TEPS consummatory pleasure and follow-up P-CAN scores worsened slightly. Negligible changes were observed in the WL control group for all reward measures ($ds < 0.20$) except for RSAS social anhedonia, which slightly worsened ($d = 0.39$). Thus, there was some indication that certain reward indices may improve following PAT-AN. However, because other reward measures either did not improve or worsened during treatment, and the pilot study sample size was small, it is difficult to determine the impact of PAT-AN on reward responding without further investigation.

B. Rationale for Inclusion and Exclusion Criteria

Inclusion criteria

All participants will be >18 years old. There is evidence that there may be different reward processes that maintain anorexia nervosa (AN) in childhood and adolescence compared to adulthood [54,55]. Further, treatment needs may differ in these different developmental stages [56]. Therefore, only adults will be enrolled in this study. Because this study involves administration of a number of interviews, questionnaires, and neurocognitive tasks that have been validated in English, only participants with the ability to read and speak in English will be included in this study.

All participants will be required to meet DSM-5 diagnosis of AN or atypical AN [1] at admission to higher-level care. Although we originally intended for the sample to only include individuals with AN, significant evidence has since emerged suggesting that atypical AN and AN are largely similar in terms of presentation, morbidity, and treatment response [52]. The Mini International Neuropsychiatric Interview [40] will be used to establish the DSM-5 diagnosis of AN or atypical AN at entry to higher level of care. BMI criteria for full syndrome AN will be <18.5 kg/m^2. However, to ensure that participants have made sufficient clinical progress in higher-level care to be classified at being at the post-acute stage, they will be required to have a current BMI ≥ 17.5 kg/m^2. Of note, originally investigators had planned for current BMI criteria to be ≥18.5 kg/m^2; however, upon further consideration expanded this criterion to accommodate the many individuals discharging prior to full weight restoration, despite substantial weight gain. Additionally, BMI ≥ 17.5 kg/m^2 is considered a standard criterion for establishing a medically safe weight for outpatient treatment [4]. Further, weight gain will need to have been a goal of treatment and the AN participants will be required to have attained a BMI increase of >0.5 kg/m^2 during acute care. Individuals with atypical AN will not be required to meet this latter criterion, due to insufficient information available about what weight goals are most appropriate for this group in higher-level care [57]. Although certain medications (e.g., dopamine reuptake inhibitors) impact reward circuitry [58], we will not exclude participants who are on any medications in order to maintain external validity with the target population. However, we will record

which medications participants are taking and will exploratorily examine whether individuals receiving medications impacting reward targets differ in their response to either treatment (see Supplement E).

Because of the experimental nature of this intervention and the fact that it is delivered remotely, we will require that all participants identify a primary physical or mental health provider outside of the treatment study who will be responsible for making ultimate decisions about appropriate type and level of treatment for the participant. To keep research and clinical decisions separate, clinical decisions will be made by the identified provider who is not part of the research team. This approach was successful in our PAT-AN pilot to ensure participant safety and oversight [29]. Specifically, participants will be required to: (a) identify a physical or mental health provider (e.g., licensed physician, nurse practitioner, psychiatrist, psychologist, social worker, marriage and family therapist) who will serve as primary provider throughout the study; and (b) sign a Release of Information that gives the study team access to discuss any change in clinical stability with the identified primary provider. Additionally, weekly weight and symptom assessments are vital to medical and psychiatric stability monitoring in addition to being treatment outcome measures. Thus, we will only enroll participants who are willing to comply with the weekly assessment protocol. Finally, in order to participate in the EMA assessments, a smartphone (any carrier or model) will be needed and to participate in teletherapy, participants will need access to a device on what they can engage with audio and video.

Exclusion criteria

Physical morbidity is a common phenomenon in AN due to the sequelae of starvation and other harmful weight-control practices (e.g., self-induced vomiting) [2]. We wish to ensure that the study participants are able to safely engage in an outpatient treatment study prior to enrolling. Therefore, we will require confirmation of medical stability for outpatient treatment prior to participants enrolling, as described below (Supplement C). Further, because BMI is a primary outcome from this study, we will exclude individuals who are pregnant, for whom weight patterns are expected to differ. We will also exclude individuals who are receiving highly overlapping treatment with PAT-AN (i.e., PAT for depression or anxiety, behavioral activation). However, we will permit enrollment of individuals who have received highly overlapping treatment with PAT-AN in the past. Given that PAT is a newer treatment [17], we expect few participants to have previously received this specific treatment. Further, PAT-AN has been significantly adapted to address the specific concerns of AN [29]. Therefore, even if participants have previously received PAT for depression or anxiety, we expect much of the content received in PAT-AN to be novel. Finally, we expect randomization to evenly distribute individuals with prior experiences with treatments similar to PAT. However, we will measure past psychological treatments at baseline and will exploratorily examine whether they impact treatment response in this trial.

Due to the effects of psychosis and mania upon reward function and treatment engagement [59,60], individuals with lifetime primary psychotic or bipolar-I disorders will be excluded. It is not clear whether a treatment enhancing positive affect would be appropriate for individuals with a bipolar-I diagnosis (who may be more likely to experience excess reward responding). Primary psychosis may interfere with the ability to participate meaningfully in the treatment sessions. Substance use disorder within the past three months will also be excluded, due to the need for specialized treatment for this population.

C. Procedures for Establishing and Monitoring Medical Stability

To evaluate initial medical stability, potentially eligible AN participants will be mailed materials to remotely measure vitals (i.e., temperature, pulse, orthostatic blood pressure), height, and blind weight using standardized procedures under the virtual supervision of research staff. Participants will then undergo a remote medical screening with a medical professional (i.e., nurse, physician), who will use their vitals information and medical history to determine potential suitability for outpatient treatment. Alternatively,

participants may provide the research team with same information obtained through an appointment with a local medical provider. We will use standard criteria to guide decisions pertaining to medical stability [4], but ultimate decisions about outpatient stability will be made at the discretion of the medical professionals performing and overseeing the screening. Individuals with atypical AN at admission to higher level care will not be required to undergo initial medical screening.

Participant safety will be monitored throughout the study to determine ongoing appropriateness for remote outpatient treatment. Similar to our pilot study [29], criteria that may flag consideration for withdrawal include: (1) acute weight loss (e.g., >0.5 lbs. per week over 4 consecutive weeks or >4 lbs. over the course of 4 weeks); (2) changes in psychiatric status that indicate a need for a higher level of care or that cause ineligibility (e.g., manic episode); (3) evidence of medical compromise indicating that outpatient care is no longer appropriate; or (4) return to higher-level care. Participants may also be withdrawn if they cannot comply with weekly weight measurements, or if they miss three consecutive sessions unless there is a reason to necessitate these misses that would not otherwise warrant withdrawal (e.g., family emergency). If a participant is under consideration for study withdrawal, their case will be reviewed by an independent safety monitor, who will not have any conflicting roles in the study protocol and will provide a recommendation about whether to retain or withdraw the participant. Withdrawn individuals will be referred to other clinical services and encouraged to complete assessments, consistent with an intent-to-treat approach, if deemed safe and ethical.

D. Expanded Measures Information

Supplemental Table 1 details the full assessment battery and the purpose of each individual measure.

Supplemental Table 1. Summary of key measures and their associated constructs.

Measure	Variable	Construct
**Outcome Variables: Acceptability**		
TAFSQ	Acceptability, feasibility, and satisfaction ratings	Treatment acceptability, feasibility, and satisfaction
CAQ	Post-session feedback ratings	Treatment component acceptability
Treatment completion	Percentage of sessions completed	Treatment acceptability
**Outcome Variables: Eating Disorder Symptoms**		
BMI	Objective height and weight	Body weight
EDE	Global score	Eating disorder symptoms (major timepoints)
CHEDS	Total score	Eating disorder symptoms (weekly)
**Outcome Variables: Affective Symptoms**		
DASS	Depression subscale score	Depressive symptoms
	Anxiety subscale score	Anxiety symptoms
C-SSRS	Presence of suicidal ideation and 3-month severity	Suicidal ideation
**Mechanistic Variables: General Reward Responsivity**		
PANAS	Positive subscale score	General reward sensitivity/positive affect
TEPS	Anticipatory subscale score	General reward anticipation
	Consummatory subscale score	General reward experiencing
WebSurf Task	Delay threshold	General reward anticipation
	Average enjoyment rating	General reward experiencing
EMA Protocol	PANAS—Positive increase following non-eating disorder events	General reward sensitivity

Supplemental Table 1. *Cont.*

Measure	Variable	Construct
**Mechanistic Variables: Eating Disorder Reward Responsivity**		
P-CAN	Pros subscale score	Eating disorder-specific reward sensitivity
Food Choice Task	Choice rating of low-fat foods	Eating disorder-specific reward anticipation
	Taste rating of low-fat foods	Eating disorder-specific reward experiencing
EMA Protocol	PANAS Positive increase following weight-control behaviors	Eating disorder-specific reward sensitivity

Note: BMI = Body Mass Index; CAQ = Component Acceptability Questionnaire [35]; CHEDS = Change in Eating Disorder Symptoms [36]; C-SSRS = Columbia—Suicide Severity Rating Scale [37]; DASS-21 = Depression, Anxiety, and Stress Scale—21-Item Version [38]; DASS-9 = Depression, Anxiety, and Stress Scale—9 Item Version [44]; EDE = Eating Disorder Examination [39]; EMA = Ecological Momentary Assessment; PANAS = Positive and Negative Affect Schedule [41]; P-CAN = Pros and Cons of Anorexia Nervosa Scale [42]; TAFSQ = Treatment Acceptability, Feasibility, and Satisfaction Questionnaire [35]; TEPS = Temporal Experiences of Pleasure Scale [43].

Eligibility

The MINI is a brief semi-structured interview that assesses symptomatology for major DSM-5 psychiatric diagnoses [40]. For the current study, the section of the MINI assessing AN will be adapted to align with DSM-5 criteria for BMI (which will be objectively measured and calculated as described below) [1]. The MINI will be administered at baseline to establish eligibility and clinically characterize the sample.

Feasibility, acceptability, and satisfaction

Treatment completion will be defined based on our previous trials [35,61,62] and established standards as attending > 80% of sessions (20–24 sessions). We will also measure number of sessions completed and examine this variable continuously to determine if number of sessions completed differs between the two treatment groups.

The Treatment Acceptability, Feasibility, and Satisfaction Questionnaire (TAFSQ), a measure adapted from our prior trials [35,61,62], will assess acceptability of PAT-AN and PBT at mid-treatment and end-of-treatment. Participants will choose which modules they find the most helpful, motivating, challenging, beneficial, and fulfilling, rate the helpfulness and difficulty of various treatment components (e.g., “Working with my therapist to understand the foundations of positive emotion”) on a scale from 1 to 5, and provide feedback for their therapist and the overall treatment program.

The Component Acceptability Questionnaire (CAQ), a measure modified from our prior trials [35,61,62], will assess treatment acceptability after each weekly session. Participants will rate helpfulness of specific components of PAT-AN (e.g., behavioral activation, mindfulness) and PBT (e.g., psychoeducation, food monitoring) and suggest possible modifications.

Eating disorder symptoms

The Eating Disorder Examination (EDE) [39] is a semi-structured, investigator-based interview that assesses eating disorder psychopathology. The EDE consists of four subscales (Restraint, Eating Concern, Shape Concern, and Weight Concern) and measures the frequency of binge eating and compensatory behaviors. Interviewers rate each item on a scale of 0 to 6, with higher scores indicating greater symptom severity. The EDE will be administered at all major study visits, and the EDE Global score will be the primary measure of eating disorder symptom severity. The EDE has well-established psychometric properties [63].

The Change in Eating Disorder Symptoms Scale (CHEDS) [36] is a 35-item self-report questionnaire designed to measure eating disorder symptoms over a weekly timespan. Participants rate the frequency of each symptom on a Likert scale of 0 = "Never" to 4 = "Always" over the past week. The CHEDS will be administered before each weekly treatment session to assess fine-grained changes in eating disorder symptoms throughout treatment.

Affective symptoms

The Depression, Anxiety, and Stress Scale—21 Item Version (DASS-21) [38] is a 21-item self-report questionnaire that assesses depression, anxiety, and stress over the past week. Total scores range from 0 to 63, with higher scores indicating greater symptom severity. The DASS-21 will be the primary measure of comorbid depressive and anxiety symptoms and will be administered at all major study visits. The DASS-9, an abridged, 9-item version with established validity and reliability [44], will be administered weekly before each treatment session to assess continuous changes in depressive and anxiety symptoms throughout treatment.

The Columbia Suicide Severity Rating Scale (C-SSRS) [37] is an interview-based measure assessing suicidal ideation and behavior. The C-SSRS will be the primary measure of suicidality for this study. We will administer the "Lifetime" and "Past 3 Months" versions at baseline and the "Since Last Visit" version at all subsequent major study visits. The C-SSRS Screener, a self-report version of the measure, will be administered weekly during treatment to capture more fine-grained changes in suicidality over the course of treatment. Past three-month suicide intensity ratings on the C-SSRS will serve as an outcome measure for baseline, mid-treatment, end-of-treatment, and follow-up time points. To investigate week-to-week changes in suicidality, we will derive a binary variable (i.e., whether suicidal ideation is present vs. not present) from the brief screening version of the C-SSRS.

Reward measures

The PANAS [41] is a 21-item self-report questionnaire comprising two subscales that measure positive and negative affect respectively. Each item is a Likert scale ranging from 1 = "Not at all" to 5 = "Very much" with higher scores indicating greater positive affect or negative affect respectively. The psychometrics of the PANAS are well-established and this measure has been used frequently to detect fine-grained changes in affect in relation to ED symptoms [64]. The PANAS will be administered before each weekly treatment session. We will use the PANAS positive affect subscale to measure self-reported affect and reward sensitivity.

The PCAN [42] is a self-report measure designed to evaluate how individuals with AN perceive the positive and negative consequences of their eating disorder. The PCAN positive subscale will be administered at all major study visits to measure changes in eating disorder-specific reward sensitivity.

The TEPS [43] is an 18-item self-report measure with two subscales evaluating the anticipatory and consummatory values of rewarding experiences. The TEPS has demonstrated strong temporal stability and validity [43]. The TEPS will be administered at all major study visits to assess general reward anticipation and experiencing.

Ecological momentary assessment (EMA)

Participants will complete one week of EMA at baseline and end-of-treatment. In line with prior research [47,64], we will collect three types of recordings: (1) signal-contingent recordings, in response to 6 semi-random signals per day; (2) time-contingent recordings, within an hour of waking up and going to sleep; and

(3) event-contingent recordings, when a participant engages in an eating episode or weight-control behavior. Bonus payments will be given for >75% response rate to incentivize compliance.

At each recording, participants will report items that will allow us to assess symptoms, putative treatment mechanisms, and their surrounding context, including the following classes of validated items derived from prior EMA research: (a) weight-control and other problematic behaviors: assessing the occurrence of meal skipping, restrictive eating, purging, and driven exercise [47,64]; (b) non-eating disorder positive life events: assessing a variety of daily positive life events, including social, relaxation, low-effort/leisure, creative, and mastery activities [65,66]; (c) positive and negative affect: assessing affect using items from the PANAS questionnaire [41]; and (d) life events and psychological experiences: similarly to our research groups' prior EMA studies [47,64], we will ask a series of questions designed to understand the external and emotional context surrounding mood and disordered behaviors (e.g., stressors, coping abilities). The full EMA protocol for this study can be found in Supplement F.

These items will allow us to assess positive affect changes from before to after eating disorder- and non-eating disorder-related events. Positive affect increases from before to after non-eating disorder positive events will assess general reward sensitivity, while positive affect increases from before to after weight-control behaviors will assess eating disorder reward sensitivity. Other events (e.g., problematic behaviors such as non-suicidal self-injury, stressful life events) will be captured to examine exploratorily and in future secondary analyses.

Neurocognitive tasks

Participants will complete two neurocognitive tasks at baseline, mid-treatment, and end-of-treatment to measure reward-based decision-making in general and weight-control contexts. Both tasks will be administered remotely through an online task repository.

The Web-Surf Task assesses reward-based decision-making related to non-eating disorder related choices. This task involves "surfing" through 4 video galleries (e.g., kittens, dance, landscape) that have been established as rewarding for healthy participants [45] in a limited time economy with random time delays assigned to each video. Participants decide which videos to watch based on video category and the time delay to access the video, and then watch the selected videos. Participants will be informed that they will have 30 minutes to choose between videos in 4 categories and that there will be a randomly selected time delay before any video. Upon presentation of a video option, a delay time will be displayed. Participants will be given the option to stay and wait for the video or skip and continue to the next gallery. If participants decide to stay on the video, this video will be presented for 4 seconds after the indicated time delay. Participants will then rate the video using a 4-star system. If a participant decides to skip the video, they go to the next gallery, which presents a new video option with a newly selected random delay. The average delay threshold, or average amount of time a participant is willing to wait to access an enjoyable video will serve as a measure of non-eating disorder reward anticipation. The average enjoyment rating after a participant has watched a video will serve as a measure of non-eating disorder reward experiencing. This task will take 30 minutes to complete.

The Food Choice task assesses reward-based decision-making in response to restrictive eating cues [46]. This task involves rating and selecting between foods ranging in caloric density and percentage from fat, specifically "high-fat" foods (>30% of calories from fat) and "low-fat" foods (<30% of calories from fat). This task has three blocks: (1) Health block; (2) Taste block; and (3) Choice block. During Health and Taste blocks, participants provide ratings of how healthy and tasty they believe each food to be on a 5-point scale. Prior research has demonstrated that individuals with AN rated low-fat foods as more tasty than healthy participants, suggesting altered reward processing related to low-fat food cues [46]. Thus, taste rating will be used as a measure of weight-control-related reward experiencing on this task. After the health and taste

ratings, a food item rated as “Neutral” on both scales is selected as the reference item. In the Choice block, participants will be instructed to choose the item they would like to consume between this neutral reference item and a series of high- and low-fat foods. Prior research has consistently demonstrated that individuals with AN select more low-fat foods during the Choice phase [67,68]. Thus, choice rating will be used as a measure of weight-control related reward anticipation on this task. This task will take 30 minutes to complete.

Assessment training and fidelity

For all interview measures (i.e., MINI, EDE, C-SSRS), research staff will attend a training led by a Ph.D.-level psychologist, observe two interviews conducted by a trained member of the research team, and be observed while conducting two interviews before they are permitted to administer the interviews on their own. Interviews completed during this training period will be co-rated by a fully trained research staff member to clarify scoring guidelines and establish reliability. All interview assessments will be administered by study staff who are kept blind to subject treatment assignment to avoid expectation biases. A random sample of 20% of recorded interviews will be selected for rating by a second blind assessor. In addition, study staff will attend weekly supervision pertaining to these assessments.

E. EMA Protocol

Below is the full EMA protocol for participants in this study. Of note, some of the items (e.g., pertaining to emotion regulation strategies, effort, and fatigue) do not pertain directly to the proposed analyses for this study, but have been included to support investigations on other topics. However, for the sake of thoroughness, all administered EMA items are included.

Beginning of the day (Time-contingent recordings)

1. Last night, how long (in minutes) did it take you to fall asleep? (in minutes)
2. Last night, how many hours of actual sleep did you get? (in hours)
3. Last night, how would you rate your sleep quality overall?
    a. Very good
    b. Fairly good
    c. Fairly bad
    d. Very bad
4. What is your level of fatigue right now? (0–10)
5. What is your level of pain right now? (0–10)
6. What is your level of stress right now? (0–10)
7. How well do you feel that you can cope with things right now? (0–10)
8. Which of these behaviors are you PLANNING on doing today (check all that apply)?
    a. Restricting your eating
    b. Skipping a meal
    c. Eating as little as possible
    d. Exercising
    e. Purging
    f. Binge eating
    g. Drinking alcohol
    h. Using recreational drugs
    i. Harming yourself on purpose

Signal-contingent recordings

1. Rate your CURRENT mood by responding to the following items (1 = not at all to 5 = extremely):
   a. Afraid
   b. Excited
   c. Relaxed
   d. Sad
   e. Proud
   f. Confident
   g. Calm
   h. Guilty
   i. Nervous
   j. Strong
   k. Enthusiastic
   l. Ashamed
   m. Determined
   n. Disgusted
   o. Happy
   p. Angry with Self
   q. Anxious
   r. Lonely
   s. At ease
   t. Irritable
2. What is your level of fatigue right now (0–10)
3. What is your level of pain right now? (0–10)
4. What is your level of stress right now (0–10)
5. How well do you feel that you can cope with things right now? (0–10)
6. How physically demanding is your current activity? (0–10)
7. How mentally demanding is your current activity? (0–10)
8. How emotionally demanding is your current activity? (0–10)
9. Are you currently around other people?
   a. YES
   b. NO
10. [If yes] Who are you currently with?
    a. Friend(s)
    b. Family
    c. Coworker(s)
    d. Classmate(s)
    e. Acquaintance(s)
    f. Stranger(s)
    g. Other
11. Since your last recording, have you experienced any of the following?
    a. Had a disagreement with someone?
    b. Were disappointed by someone?
    c. Were reminded of something painful from the past?
    d. Viewed upsetting social media?
    e. Were rejected or ignored by others?

   f. Did not hear from someone you expect to hear from?
   g. Experienced unwanted physical contact (crowded, pushed)?
   h. Experienced problems with friends or family members?
   i. Were forced to socialize?
   j. Experienced problems with school/work?
   k. Were exposed to a feared situation?
   l. Received bad news?
   m. Experienced stigma due to some aspect of your identity?

12. How stressful was it when you [INSERT FROM ABOVE] (1 = not at all to 5 = very much)?
13. [If stigma endorsed] In your opinion, what was the reason you were stigmatized?
    a. My weight
    b. My sexual orientation
    c. My gender
    d. My race
    e. My ethnicity
    f. My age
    g. My social class
    h. My religion
    i. Other
14. Since your last recording, which of the following have you engaged in?
    a. Work
    b. School work
    c. Traveling between places
    d. Chores or cleaning
    e. Volunteer activity
    f. Social activity
    g. Relaxing/leisure
    h. Spending time with family
    i. Social media/computer entertainment
    j. Playing a game
    k. Religious activity
    l. Sports or physical activity
    m. Watching TV or a movie
    n. Creative activities
    o. Hobbies or crafts
    p. Reading or writing for enjoyment
    q. Other recreation
15. How enjoyable was it when you engaged in [INSERT FROM ABOVE] (1 = not at all to 5 = very much)?
16. Since your last recording, have you used any of these strategies to try to manage your emotions?
    a. Thinking differently about the situation
    b. Practicing acceptance
    c. Practicing gratitude
    d. Problem-solving
    e. Engaging in pleasant activities
    f. Thinking about past or future pleasant activities
    g. Noticing the positive aspects of your situation

h. Practicing generosity or kindness towards yourself
i. Taking ownership of positive experiences
j. Suppressing your thoughts or emotions
k. Thinking repeatedly about your emotions
l. Engaging in impulsive behaviors
m. Avoiding negative emotions

17. How did [INSERT FROM ABOVE] make you feel? (1 = much worse to 5 = much better)?
18. Since your last recording, which of these behaviors did you engage in?
    a. I restricted
    b. I binged
    c. I purged
    d. I exercised
    e. I skipped a meal
    f. I drank alcohol
    g. I used recreational drugs
    h. I harmed myself on purpose
    i. None of the above
19. Did you previously record that you [INSERT FROM ABOVE]?
    a. Yes
    b. No
20. How long ago did you last [INSERT FROM ABOVE]?
    a. Less than 15 minutes
    b. 15 minutes
    c. 30 minutes
    d. 45 minutes
    e. 60 minutes
    f. 75 minutes
    g. 90 minutes
    h. 2 hours
    i. More than 2 hours
21. [If exercise] How long did you exercise?
    a. 1 = <10 minutes
    b. 2 = 10–20 minutes
    c. 3 = 30–40 minutes
    d. 4 = 40–50 minutes
    e. 5 = 50–60 minutes
    f. 6 = >60 minutes
22. Since last signal, have you eaten any of the following?
    a. Snack
    b. Meal
    c. Binge
    d. None of the above
23. Did you previously record the [INSERT FROM ABOVE]?
    a. Yes
    b. No
24. How long ago did you eat the [INSERT FROM ABOVE]?

   a. Less than 15 minutes
   b. 15 minutes
   c. 30 minutes
   d. 45 minutes
   e. 60 minutes
   f. 75 minutes
   g. 90 minutes
   h. 2 hours
   i. More than 2 hours

25. Check all that apply to this eating episode:
    a. I limited calories
    b. I limited fat
    c. I limited carbs
    d. I ate as little as possible
    e. I felt out of control
    f. I ate an excessive amount of food
    g. None of the above

Record a behavior (*Event-contingent recordings)*

1. Which of these behaviors did you engage in?
   a. I restricted
   b. I binged
   c. I purged
   d. I exercised
   e. I skipped a meal
   f. I drank alcohol
   g. I used recreational drugs
   h. I harmed myself on purpose
   i. None of the above
2. How long ago did you [INSERT FROM ABOVE]?
   a. Less than 15 minutes
   b. 15 minutes
   c. 30 minutes
   d. 45 minutes
   e. 60 minutes
   f. 75 minutes
   g. 90 minutes
   h. 2 hours
   i. More than 2 hours
3. [If exercise] How long did you exercise?
   a. 1 = <10 minutes
   b. 2 = 10–20 minutes
   c. 3 = 30–40 minutes
   d. 4 = 40–50 minutes
   e. 5 = 50–60 minutes
   f. 6 = >60 minutes

4. Rate your CURRENT mood by responding to the following items (1 = not at all to 5 = extremely):
    a. Afraid
    b. Excited
    c. Relaxed
    d. Sad
    e. Proud
    f. Confident
    g. Calm
    h. Guilty
    i. Nervous
    j. Strong
    k. Enthusiastic
    l. Ashamed
    m. Determined
    n. Disgusted
    o. Happy
    p. Angry with Self
    q. Anxious
    r. Lonely
    s. At ease
    t. Irritable
5. What is your level of fatigue right now (0–10)
6. What is your level of pain right now? (0–10)
7. What is your level of stress right now (0–10)
8. How well do you feel that you can cope with things right now? (0–10)
9. How physically demanding is your current activity? (0–10)
10. How mentally demanding is your current activity? (0–10)
11. How emotionally demanding is your current activity? (0–10)

Record an eating episode (*Event-contingent recordings)*

1. How would you categorize this eating episode?
    a. Meal
    b. Snack
    c. Binge
    d. Other (e.g., hard candy, gum, mint)
2. How long ago did you eat the _____?
    a. Less than 15 minutes
    b. 15 minutes
    c. 30 minutes
    d. 45 minutes
    e. 60 minutes
    f. 75 minutes
    g. 90 minutes
    h. 2 hours
    i. More than 2 hours
3. [If not a binge episode] Check all that apply to this eating episode:

   a. I limited calories
   b. I limited fat
   c. I limited carbs
   d. I ate as little as possible
   e. I felt out of control
   f. I ate an excessive amount of food
   g. None of the above
4. [If a binge episode] Check all that apply to this eating episode:
   a. I felt out of control
   b. I felt driven/compelled to eat
   c. I ate an excessive amount of food
   d. None of the above
5. Rate your CURRENT mood by responding to the following items (1 = not at all to 5 = extremely):
   a. Afraid
   b. Excited
   c. Relaxed
   d. Sad
   e. Proud
   f. Confident
   g. Calm
   h. Guilty
   i. Nervous
   j. Strong
   k. Enthusiastic
   l. Ashamed
   m. Determined
   n. Disgusted
   o. Happy
   p. Angry with Self
   q. Anxious
   r. Lonely
   s. At ease
   t. Irritable
6. What is your level of fatigue right now (0–10)
7. What is your level of pain right now? (0–10)
8. What is your level of stress right now (0–10)
9. How well do you feel that you can cope with things right now? (0–10)
10. How physically demanding is your current activity? (0–10)
11. How mentally demanding is your current activity? (0–10)
12. How emotionally demanding is your current activity? (0–10)

End of the day (Time-contingent recordings)

1. Rate your CURRENT mood by responding to the following items (1 = not at all to 5 = extremely):
   a. Afraid
   b. Excited
   c. Relaxed

   d. Sad
   e. Proud
   f. Confident
   g. Calm
   h. Guilty
   i. Nervous
   j. Strong
   k. Enthusiastic
   l. Ashamed
   m. Determined
   n. Disgusted
   o. Happy
   p. Angry with Self
   q. Anxious
   r. Lonely
   s. At ease
   t. Irritable
2. What is your level of fatigue right now? (0–10)
3. What is your level of pain right now? (0–10)
4. What is your level of stress right now? (0–10)
5. How well do you feel that you coped with things today? (0–10)
6. How physically demanding was your day? (0–10)
7. How mentally demanding was your day? (0–10)
8. How emotionally demanding was your day? (0–10)
9. Since waking up this morning, did you engage in any of the following?
   a. I went for 8 waking hours without eating
   b. I limited daily intake to less than 1200 calories

F. Additional Statistical Information

Exploratory covariates

In order to reduce the number of variables in our model and, thereby, preserve power, we will not include any covariates in our primary analyses. However, we will conduct secondary exploratory analyses examining the influence of key demographic variables (i.e., age, sex, gender, race, ethnicity, baseline BMI) and clinical variables (i.e., AN subtype, duration of illness, type of higher-level care, time since discharge from higher-level care, engagement in other concurrent therapies, use of psychotropic medications, prior treatments overlapping with PAT-AN) on study outcomes. Potential covariates will first be compared between groups to determine if treatments varied on these indices. All planned analyses will then be repeated in an exploratory manner with the inclusion of any items that differed between treatments as potential covariates. This will provide initial information about if any results from the trial may be accounted for by group differences on these key variables.

Power analyses

Our power analyses calculate the minimum detectable effect size for $N = 80$ ($n = 40$/group) for the primary study sample and a stationary first-order autoregressive coefficient of 0.5 for all repeated measures. All analyses assume a conservative dropout rate across conditions of 30% at end-of-treatment and 3-month

follow-up with a target final sample size of 56, which was used as the final sample size for all power analyses. For Aim 1, our sample size will give us 80% power to detect a moderate effect $f^2 = 0.15$ on acceptability and a moderate effect size of *OR* = 2.50 for group on attrition outcomes. We will have 80% power to detect a moderate effect size of $f^2 = 0.21$ on eating disorder symptoms, BMI, affective symptoms, and suicidality across the 3 main time points. In our pilot study, effects sizes on clinical outcomes were generally medium to large [29]. For Aim 2, our sample size will give us 80% power to detect a medium effect size of $f^2 = 0.20$ on primary mechanistic targets across the 3 main time points. For Aim 3, our sample size will give us 80% power to detect a moderate effect size $f^2 = 0.27$ of primary mechanistic targets across two time points (baseline, end-of-treatment) in predicting outcomes at two time points (end-of-treatment, follow-up).

Missing data

We will attempt to obtain outcome measures from all participants on the indicated timescale even in the case of treatment non-completion or investigator-initiated withdrawal. Our primary approach will be an intent-to-treat model in which all available data are included in outcome comparisons. As a secondary approach, we will compare all treatment completers (i.e., those completing > 80% of sessions) [69]. Item-level missing data will be imputed using the Multiple imputation by chained equations (MICE) package [70]. Imputation will be conducted on all items within the dataset relevant to the planned primary and exploratory analyses, including potential covariates, with the exception of demographic variables and variables involving skip logic (i.e., data will not be imputed for variables where missingness is meaningful within the structure of the assessment). In line with current recommendations, the number of imputations will be derived from the average rate of missingness across the dataset (e.g., if 10% of data are missing, 10 imputations will be conducted) [71]. Sensitivity analyses will be conducted using three methods for handling missing data: analyses performed on all available data (primary), multiple imputation, and completer analysis using only those cases with complete data. Results will be compared for consistency across the three methods.
